# Generalised framework for multi-criteria method selection: Rule set database and exemplary decision support system implementation blueprints

**DOI:** 10.1016/j.dib.2018.12.015

**Published:** 2018-12-12

**Authors:** Jarosław Wątróbski, Jarosław Jankowski, Paweł Ziemba, Artur Karczmarczyk, Magdalena Zioło

**Affiliations:** a Faculty of Economics and Management, University of Szczecin, Mickiewicza 64, 71-101, Szczecin, Poland; b Faculty of Computer Science and Information Systems, West Pomeranian University of Technology, Zolnierska 49, 71-210 Szczecin, Poland

## Abstract

This data article describes the analysis of 56 MCDA (Multi-Criteria Decision Analysis) methods in regards to 9 decision-making problem characteristics structured into 3 levels. The impact of uncertainty in decision-making problem description on MCDA method selection is studied. 450,000 possible descriptions of a decision problem were studied, resulting in sets of rules which can serve as input to uncertainty-aware MCDA method selection decision support systems. Comprehensive analyses of the obtained rule sets are provided. An exemplary decision support system based on the presented data was created and is available at http://www.mcda.it. Moreover the technical documentation needed to create and expand such system is provided in this data article. The data and system can be easily extended and the authors invite all researchers to contribute.

**Specifications table**

**Table:** 

Subject area	*Economics, Computer Science*
More specific subject area	*MCDA, Decision Support*
Type of data	*Microsoft Excel Worksheet*
How data was acquired	*Literature analysis, computer processing*
Data format	*Both raw and analyzed*
Experimental factors	*A set of 56 MCDA methods were initially studied regarding 9 decision problem descriptors organized into 3 levels of hierarchy. The obtained data was the input for the experiment.*
Experimental features	*During the experiment, 4536 possible rules for MCDA method selection were generated, based on all non-contradictory values of the 9 decision problem descriptors structured into 3 levels of hierarchy, taking into account uncertainty. The obtained rule set was further filtered by removing the rules which returned no matching methods, resulting in 656 rules. Analyses of the aggregate data were performed.*
Data source location	*Not applicable*
Data accessibility	*The data is presented in a Microsoft Excel Worksheet, which is provided as*Supplementary data*for this article.*
Related research article	*Jarosław Wątróbski, Jarosław Jankowski, Paweł Ziemba, Artur Karczmarczyk, Magdalena Zioło, Generalised framework for multi-criteria method selection, Omega, 2018, ISSN 0305–0483,*https://doi.org/10.1016/j.omega.2018.07.004

**Value of the data**

• The data serves as an easily-expandable benchmark of MCDA methods regarding a carefully selected set of decision problem descriptors organized into 3 levels of hierarchy.
• The data provides insight into the areas of the decision-making problem space which have yet to be covered with MCDA methods.
• The data provides input for MCDA method selection decision support systems as well as blueprints for creation of such systems.
• The data facilitates the research on the influence of uncertainty on the MCDA method selection problem.

## 1 Data

The Microsoft Excel Worksheet that is provided as Supplementary data for this article contains a set of 28 sheets. The first sheet, contains a list of 56 methods and their properties regarding a decision-making problem׳s characteristics. The second and third sheets contain the sets of rules obtained during the experiment. The remaining sheets contain analyses of the two sets of rules.

Moreover, this data article comes with a set of documents which facilitated the creation of the http://www.mcda.it service:

• BPMN process (in Bizagi Modeler.bpm, as well as.png formats) describing the MCDA method selection decision-making process;
• database diagram (in MySQL Workbench.mwb, as well as.png formats) showing the table for storing MCDA methods’ properties;
• code documentation, describing how the exemplary decision support system was built and how it can be extended.

## 2 Experimental design, materials, and methods

**Fig. 1:**
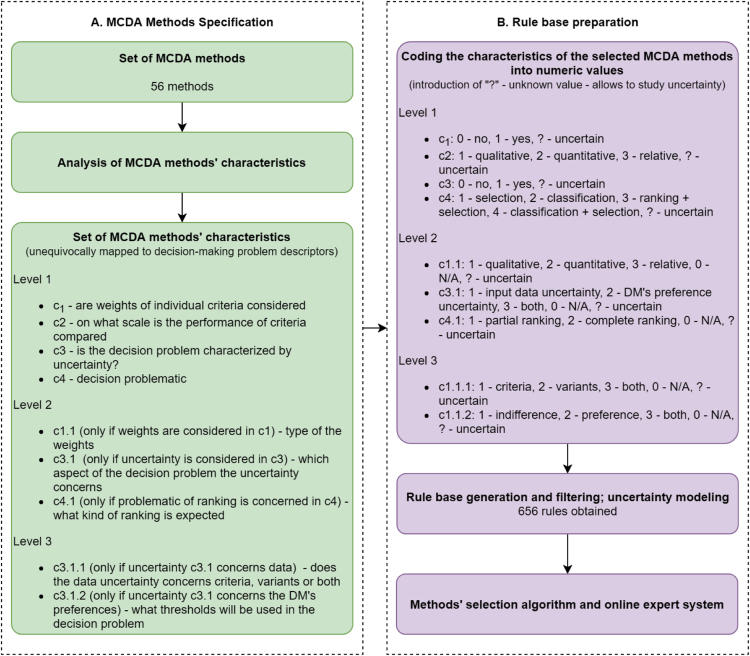
Explanation of the two parts of the experiment: A) specification of the MCDA methods, B) preparation of the rule base.

The experiment was conducted on the basis of a comprehensive literature review and was split into two steps: MCDA methods specification, followed by the rule base preparation (see Fig. 1). As a result of the first stage, 56 MCDA methods were selected and 9 MCDA methods’ characteristics (unequivocally mapped to decision-making problem descriptors) in 3 levels of hierarchy were identified (see Fig. 1A and the “1. methods-descriptors” sheet). In the second step, all the considered values of the MCDA methods’ characteristics obtained numeric values, as well as 0 (not applicable, N/A) and? (uncertain) were added for researching the uncertainty (see Fig. 1B). The obtained rule set served as the database of a MCDA method selection decision support system used during the experiment.

**Fig. 2:**
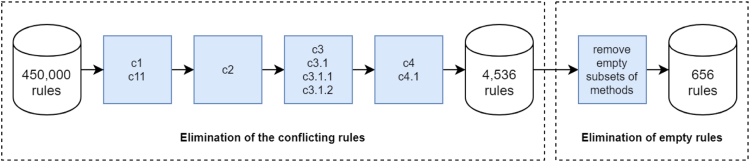
Process of obtaining the rule set during the experiment.

During the experiment, such obtained decision support system was queried 450,000 times for every possible set of known and unknown decision problem characteristics. Subsequently, the rules holding contradictory descriptor values were filtered to eliminate contradictory rules, resulting in 4,536 rules (see spreadsheet 2). Eventually, the rules returning empty sets of methods were eliminated, resulting in 656 rules (see spreadsheet 3). The described process is presented on Fig. 2.

Similar rule sets were obtained for two levels of hierarchy, as well as for a flat structure of decision problem characteristics (see spreadsheets 4–7). These sets were obtained by removing from consideration the criteria from third and second level of hierarchy and the rules depending on them.

**Fig. 3:**
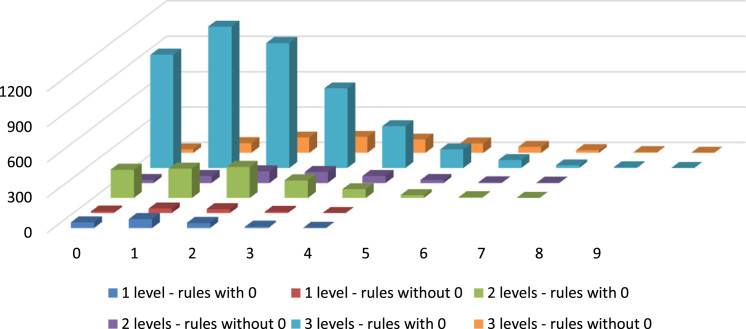
Comparison of rule sets with and without rules returning no matching methods.

Subsequently, the number of matching rules for each set of descriptors within each rule set were plotted on charts. Eventually, the differences in numbers of matched rules between rule sets with various levels of hierarchy and with or without rules returning no methods were studied and charted (see Fig. 3). The methodological assumptions which lead to the obtainment of the data presented in this data article are presented in Supplementary material.

