# Supplementary material for: Generalised framework for multi-criteria method selection: Rule set database and exemplary decision support system implementation blueprints
Source: Data Brief. 2018 Dec 12;22:639–42. doi: 10.1016/j.dib.2018.12.015 (PMC6327857; doi:10.1016/j.dib.2018.12.015)
Supplement: Supplementary file 1 — Supplementary material [file mmc1.pdf]

Conflict of Interest and Authorship Conformation Form

Please check the following as appropriate:

- All authors have participated in (a) conception and design, or analysis and interpretation of the data; (b) drafting the article or revising it critically for important intellectual content; and (c) approval of the final version.

- This manuscript has not been submitted to, nor is under review at, another journal or other publishing venue.

- The authors have no affiliation with any organization with a direct or indirect financial interest in the subject matter discussed in the manuscript

- The following authors have affiliations with organizations with direct or indirect financial interest in the subject matter discussed in the manuscript:

**Authors:**	Jarosław Wątróbski[1] Jarosław Jankowski[2] Paweł Ziemba[1]<br>Artur Karczmarczyk[2] Magdalena Zioło[1]
**Affiliations:**	[1] Faculty of Economics and Management, University of Szczecin, Mickiewicza 64,<br>71-101, Szczecin, Poland,<br>{jaroslaw.watrobski@wneiz.pl, pziemba@ajp.edu.pl,<br>magdalena.ziolo@usz.edu.pl}<br>[2] Faculty of Computer Science and Information Systems, West Pomeranian University of Technology, Zolnierska 49, 71-210 Szczecin, Poland, {jjankowski, akarczmarczyk}@wi.zut.edu.pl
