# Supplementary material for: Generalised framework for multi-criteria method selection: Rule set database and exemplary decision support system implementation blueprints
Source: Data Brief. 2018 Dec 12;22:639–42. doi: 10.1016/j.dib.2018.12.015 (PMC6327857; doi:10.1016/j.dib.2018.12.015)
Supplement: Supplementary file 2 — Supplementary material [file mmc2.zip › mcda-it-code-documentation.pdf]

Jarosław Wątróbski
Faculty of Economics and Management, University of Szczecin, Mickiewicza 64, 71-101 Szczecin, Poland

MCDA.IT

Code documentation

Introduction

This document contains information about the structure of the exemplary decision support system created as part of [1] research. It explains how the software has been built, in order to facilitate the creation of similar systems in the future.

Application Foundations

Prior to creation of the system, some fundamental assumptions were made. First and foremost, it was decided that the decision support system should be widely available, therefore it should be created as a web application. Moreover, the application should be lightweight, in order to allow deployment to free or inexpensive hosting providers. Therefore it was decided to build the system based on a lightweight interpreted PHP [2] language and free MySQL [3] database. However, creation of a website application from scratch is complex and error-prone, therefore it has been decided to build the application based on Symfony Framework 3.4 [4], a leading framework for building websites and web applications with over 3 thousand contributors, 600 thousand developers and over 48 million monthly downloads.

Project Code Structure

The project is based on the Standard Edition Distribution of the Symfony Framework [5]. The directory structure of this distribution is presented on Figure 1a. The actual application code under Symfony Framework 3.4 is required to be put into the **src** directory. As presented on Figure 1b, the code of the decision support system was packaged into a single bundle called **McdaDeciderBundle**. The conventions of the framework used require the developers to organize their software in an organized manner [6].

As part of the project, a single entity called **Method.php** was created, for storing and processing the characteristics of MCDA methods. In order to fetch the entities from database, a **MethodRepository.php** repository was created with a single method **findByCriteria()** for fetching from the database exclusively the methods having requested properties. The method takes into account the uncertainty of the decision-making situation and its algorithm is described in [1].

In order to easily manage various options of each property of MCDA methods, a set of PHP enumeration classes was created (see Figure 1c). Each of the enumeration classes implements the **EnumInterface** (see Figure 2) and derives from the **BaseEnum** class (see Figure 3 class).

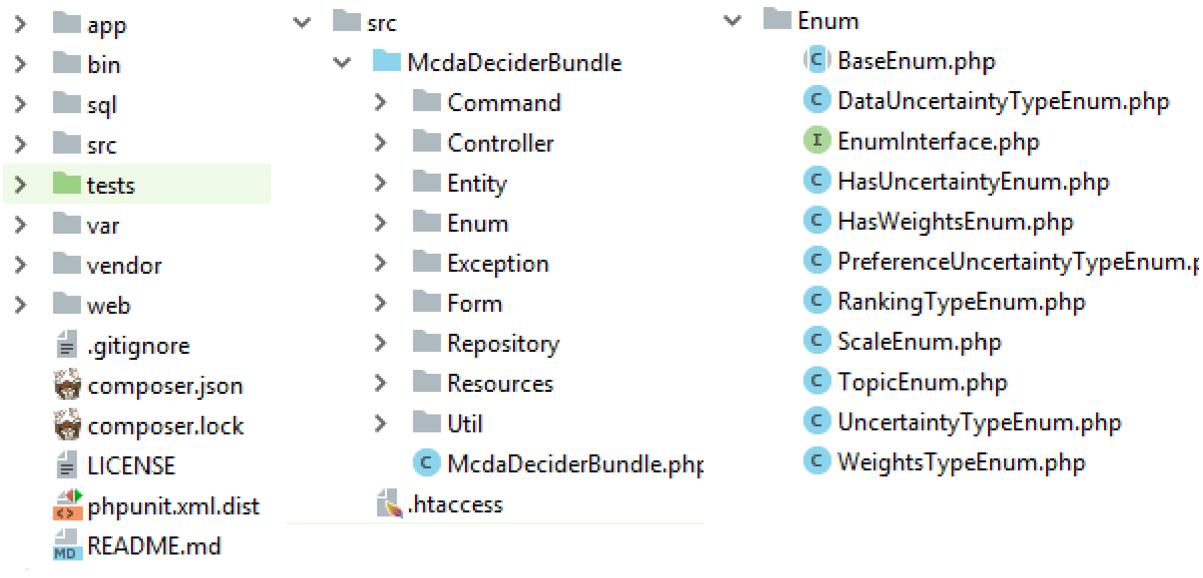

(a) (b) (c)

Figure 1 Directory structure of the project: a) basic Symfony Framework Standard Edition Distribution structure; b) McdaDeciderBundle structure.

Main Controller of the System

The system follows an MVC design pattern. Therefore, all logic is stored in a controller. In case of the MCDA.IT decision support system, the logic is stored in a single **src/McdaDeciderBuncle/Controller/DefaultController.php** file. The controller contains three actions:

- indexAction – for displaying the home page of the service;
- fullRangeAction – a facility action for generating sets of methods matching all possible queries; the data obtained from this action was used to perform the experimental study in [1];
- ajaxDecideAction – this is an AJAX action called by the indexAction homepage each time when the user selects any of the options of the decision problem characteristics. This action returns a single set of MCDA methods matching the characteristics provided by the user.

Possibility to Extend the System

Since the software was created with a freely available and well documented framework [4], it is easily possible to extend its possibilities by creating and adding more bundles. Moreover, the database of available methods can be easily extended by adding to the database additional methods and their characteristics. After adding new methods, the decision support system will instantly begin returning them when matching properties' values are chosen by the user.

```php
<?php
namespace McdaDeciderBundle\Enum;

use McdaDeciderBundle\Exception\InvalidEnumValueException;

interface EnumInterface
{
    public static function getChoices();

    /**
     * Gets string representation of the enum value.
     *
     * @param $value
     * @return string
     * @throws InvalidEnumValueException
     */
    public static function getStringFromValue($value);
}

```

Figure 2 Definition of the EnumInterface interface.

```php
namespace McdaDeciderBundle\Enum;


use McdaDeciderBundle\Exception\InvalidEnumValueException;

abstract class BaseEnum implements EnumInterface
{
    /**
     * @inheritdoc
     */
    public static function getStringFromValue($value)
    {
        $choices = static::getChoices();
        if (! isset($choices[$value])) {
            throw new InvalidEnumValueException("Invalid value '$value'");
        }

        return $choices[$value];
    }
}

```

Figure 3 Definition of the BaseEnum class.

References

[1] J. Wątróbski, J. Jankowski, P. Ziemba, A. Karczmarczyk, and M. Zioło, “Generalised framework for multi-criteria method selection,” *Omega*, Jul. 2018.

[2] “PHP Website.” [Online]. Available: http://php.net/. [Accessed: 18-Aug-2018].

[3] “MySQL Website.” [Online]. Available: https://www.mysql.com/. [Accessed: 18-Aug-2018].

[4] “Symfony Framework 3.4 Documentation.” [Online]. Available: http://symfony.com/doc/3.4/setup.html. [Accessed: 18-Aug-2018].

[5] “Symfony Framework Standard Edition Distribution.” [Online]. Available: https://packagist.org/packages/symfony/framework-standard-edition. [Accessed: 18-Aug-2018].

[6] “Symfony Quick Tour: The Architecture.” [Online]. Available: https://symfony.com/doc/3.4/quick_tour/the_architecture.html. [Accessed: 18-Aug-2018].
