# Supplementary figures and images for: Generalised framework for multi-criteria method selection: Rule set database and exemplary decision support system implementation blueprints
Source: Data Brief. 2018 Dec 12;22:639–42. doi: 10.1016/j.dib.2018.12.015 (PMC6327857; doi:10.1016/j.dib.2018.12.015)

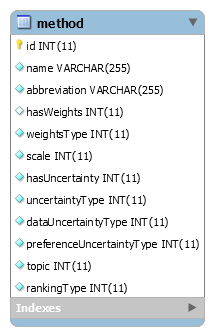

Supplement: Supplementary file 2 — Supplementary material [file mmc2.zip › mcda-it-db-diagram.png]

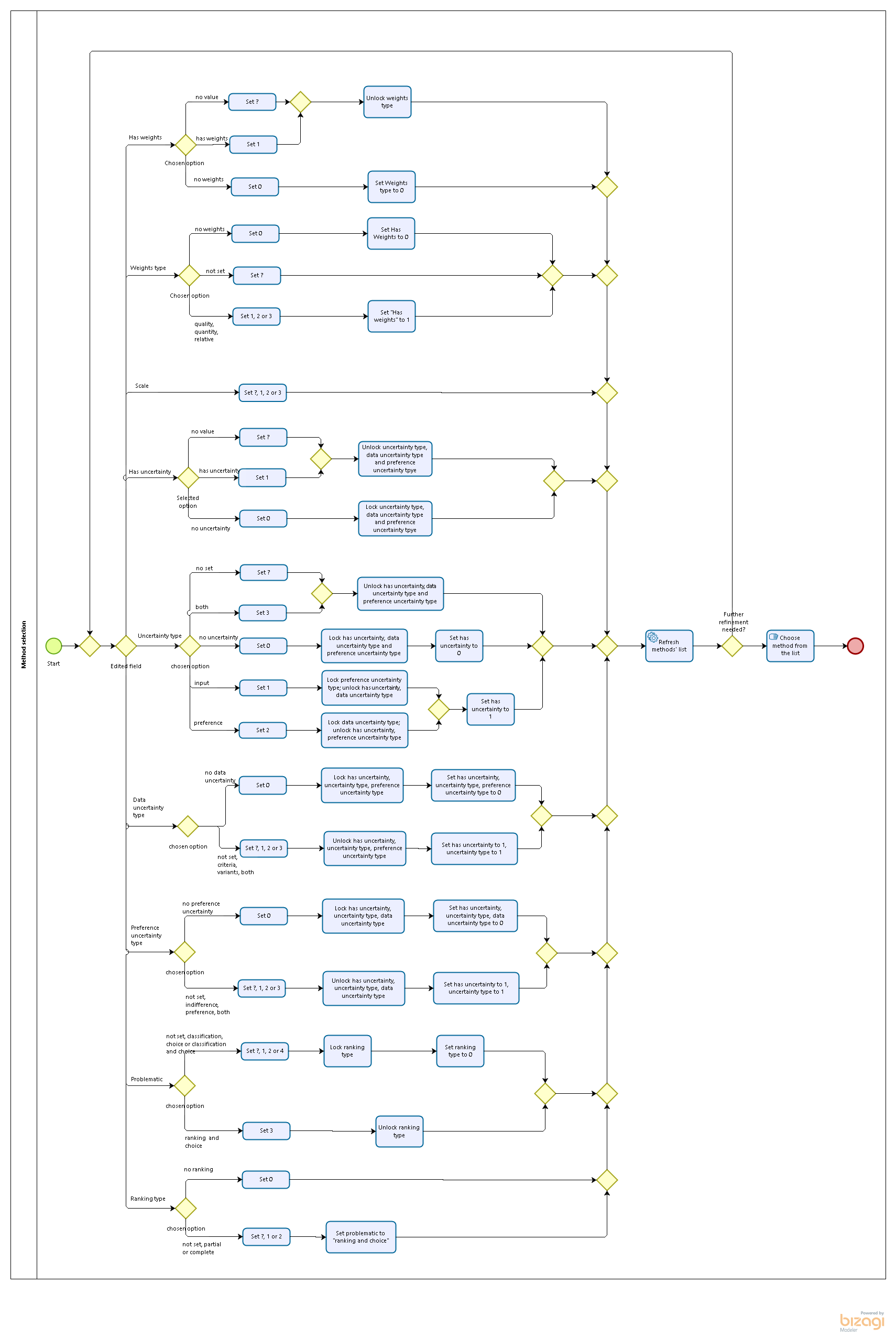

Supplement: Supplementary file 2 — Supplementary material [file mmc2.zip › mcda-decider.png]
