# Supplementary material for: Generalised framework for multi-criteria method selection: Rule set database and exemplary decision support system implementation blueprints
Source: Data Brief. 2018 Dec 12;22:639–42. doi: 10.1016/j.dib.2018.12.015 (PMC6327857; doi:10.1016/j.dib.2018.12.015)
Supplement: Supplementary file 3 — Supplementary material [file mmc3.pdf]

Supplementary material: A summary of the methodological assumptions of the framework for multi-criteria method selection [1].

This data article is based on a dataset obtained during the creation of the generalized framework for multi-criteria method selection [1]. In this section its methodological foundations are summarized for a better understanding of the presented data article. Nonetheless, it should be noted that the full paper [1] is freely available for all readers in Open Access.

Classically, a decision problem (DP) can be presented in the form of a three-element set (A, G, E), where A represents the set of possible decision variants, G denotes a set of criteria and E is the set of performance of each variant under each criterion, therefore, $E = G(A)$.

Each decision problem can be characterized by a set of decision problem descriptors (c). The authors analyzed a set of 56 MCDA methods and their combinations and, on their basis, developed a three-level hierarchy of nine decision problem descriptors (c), which indicate the required properties (m) that each MCDA method considered for solving the DP should have. On each level of the hierarchy the accuracy of the DP description is more profound. It is particularly important in case of uncertainty, i.e. when the decision maker (DM) does not have the full knowledge of the DP, and, therefore, cannot define all nine decision problem descriptors.

On the first level of the hierarchy, the authors defined three decision problem descriptors (c):

- c1 – are weights of individual criteria considered [0 – no, 1 – yes]
- c2 – on what scale is the performance of criteria compared [1 – qualitative, 2 – quantitative, 3 – relative]
- c3 – is the decision problem characterized by uncertainty [0 – no, 1 – yes]
- c4 – what is the decision problematic [1 – selection, 2 – classification + selection, 3 – ranking + selection, 4 – classification + selection]

Although it can be assumed that c2 is fully specified, the remaining descriptors can be further defined and, hence, the second level of the proposed hierarchy:

- c1.1 – what is the type of weights considered [1 – qualitative, 2 – quantitative, 3 – relative, 0 – N/A]
- c3.1 – which aspect of the decision problem the uncertainty concerns [1 – input data uncertainty, 2 – the DM's preference uncertainty, 3 – both, 0 – N/A]
- c4.1 – what kind of ranking is expected [1 – partial ranking, 2 – complete ranking, 0 – N/A]

Last, but not least, the c3.1 descriptor can be defined further:

- c3.1.1 – what does the input data uncertainty concerns [1 – criteria, 2 – variants, 3 – both, 0 – N/A]

- c3.1.2 – what preference thresholds will be used in the decision problem [1 – indifference, 2 – preference, 3 – both, 0 – N/A]

A total of 56 MCDA methods was studied regarding the aforementioned nine criteria. The authors' findings were presented in spreadsheet "1. Methods-descriptors". An example of selected five methods is presented in the table below:

Method	C1	C1.1	C2	C3	C3.1	C3.1.1	C3.1.2	C4	C4.1
**AHP**	1	3	3	0	0	0	0	3	2
**AHP+TOPSIS**	1	3	2	0	0	0	0	3	2
**ANP**	1	3	3	0	0	0	0	3	2
**ARGUS**	1	1	1	0	0	0	0	1	0
**COMET**	0	0	2	1	1	2	0	3	2

The table from spreadsheet "1. Methods-descriptors" was used to generate the database for selecting MCDA methods based on the decision problem characteristics. For better explanation of the framework, a simplified example based on the five MCDA methods above should be presented.

If the DM knew that the analyzed decision problem had the following characteristics: c1=1, c1.1=3, c2=3, c3=0, c3.1.1=0, c3.1.2=0, c4=3, c4.1=0, based on the table above it is easy to observe that only two methods are valid for using in resolving that problem: AHP and ANP.

Nevertheless, the DM's knowledge of the decision problem can be often limited. For example, if the DM does not know a single characteristic of the decision problem, i.e. c1=?, c1.1=?, c2=?, c3=?, c3.1=?, c3.1.1=?, c3.1.2=?, c4=?, c4.1=?, then it is easy to note that all 5 methods are equally valid. On the other hand, if the DM knew exclusively that in the decision problem the criteria weights should be considered, i.e. c1=1, c1.1=?, c2=?, c3=?, c3.1=?, c3.1.1=?, c3.1.2=?, c4=?, c4.1=?, then only four of the above methods would be valid, since the COMET method does not use weights of criteria. Furthermore, if the DM additionally knew that the criterial performance should be compared on a relative scale, i.e. c1=1, c1.1=?, c2=3, c3=?, c3.1=?, c3.1.1=?, c3.1.2=?, c4=?, c4.1=?, then only two methods of the above five would be valid: AHP and ANP. In a similar manner, the full space of possibilities was studied by the authors, thus producing a set of 4536 such rules. However, majority of them returned an empty set of MCDA methods, showing niches for further MCDA methods' development. A total of 656 rules returning at least one MCDA methods was obtained.

Last, but not least, the same database was used to study the differences between sets of four flat criteria (c1, c2, c3, c4), seven 2-level criteria (c1, c1.1, c2, c3, c3.1, c4, c4.1) and nine 3-level criteria (c1, c1.1, c2, c3, c3.1, c3.1.1, c3.1.2, c4, c4.1) – see spreadsheets 2-28.
